# Supplementary material for: Learning curve and surgical outcome of robotic assisted colorectal surgery with ERAS program
Source: Sci Rep. 2022 Nov 29;12:20566. doi: 10.1038/s41598-022-24665-w (PMC9709162; doi:10.1038/s41598-022-24665-w)
Supplement: Supplementary file 2 — Supplementary Information 2. [file 41598_2022_24665_MOESM2_ESM.zip › RAL Raw data-Ñ[▒K/065/HS_ñw╝╨░O▒Kñσ.pdf]

正式摘要(電子簽章：000000212852589)		
W52 / W52 ☐英文標題(注意:無英文姓名-請補病人基本檔!)	☐【讀取】憑證	

臺中榮民總醫院
出院病歷摘要

轉入號 : 轉出號 :

[redacted]

**(8)**流水編號	
**(9)**入院日期	110/03/16 (2021) 直腸外科 W52 - 112
**(10)**轉科日期	
**(11)**出院日期	110/03/23 (2021) 住院天數計 7 日

診斷

- 入院:1. Sigmoid Ca, 20 from AV (仁慈醫院), CK (+)p53 (-) mCEA (+) CDX-2 (+) EGFR(2+)
  2. DM under OHA
- 出院:1. Sigmoid Ca, 20 from AV (仁慈醫院), CK (+)p53 (-) mCEA (+) CDX-2 (+) EGFR(2+), s/p robotic AR on 2021/03/17

主訴

- sigmoid ca, cT1-2N0M0

病史

- The patient is a 48 years old male with history of
  1. DM under OHA poor control (03/090 HbA1C :7.3)
  2. HTN under medicine control
  stool OB(+) was noted during physical examination. 02/25 Colonscopy +pathology : Sigmoid Ca, 20 from AV (仁慈醫院), CK (+)p53 (-) mCEA (+) CDX-2 (+) EGFR(2+). He went to CRS OPD for further treatment , 03/10 CT showed mild wall-thickening of sigmoidal colon, cT1-2N0M0. He was admitted for ROBOTIC AR.

體檢發現

- Anal inspection:
  Recto digital examination:
  Sigmoidoscopy:
  Anoscopy:
  Abdomen:
  - General appearance :
    - easy looking , conscious clear , without cardiopulmonary distress
    - Vital signs自動帶入date 體溫(BT) 脈搏(P) 呼吸(R) 血壓(BP) 身高(BH) 體重(BW) BMI
      20210316 35.4℃ 92次/分 18次/分 119/90mmHg 169.9cm 68.9kg 23.9
    - Vital signs: B.P. 119/9092 mmHg , P.R. 92 /min , R.R. 18 /min , B.T. 35.4 ℃
  - HEENT :
    - Eyes: no anemic conjunctiva, no icteric sclera, normal EOM
    - ENT: no otorrhea, no rhinorrhea, no erythematous mucosa, no injected tonsil, no deformity
  - Neck: supple, freely movable, trachea midline,no deformity, no engorged jugular vein , no palpable thyroid or lymph node enlargement
  - Chest:
    - Inspection: Grossly normal, symmetric and fully expansion, no accessory muscle used, no operation scar, no spider angiomata, no gynecomastia
    - Palpation: no palpable mass or nodule, no subcutaneous emphysema, no tenderness
    - Ausculation: clear breathing sound
  - Heart:
    - Palpation: no thrill, no heave
    - Percussion: normal heart size

- Ausculation: regular heart beat, normal S1, S2, no gallop, no murmur
- Extremities: No deformity, full ranged of motion without limitation, no clubbing finger or nail cyanosis, no pitting edema, no ulcer or pigmentation, no obvious skin lesion, no varicose vein, no bone, joint or muscle tenderness

CRS CONDITION

- Karnofsky performance status: (100) Normal, no complaints, no evidence of disease
- PE of Abdomen:
- CEA: 8.97 ng/ml
- Imaging Studies:
- Colonoscope report: Sigmoid Ca, 20 from AV (仁慈醫院), CK (+)p53 (-) mCEA (+) CDX-2 (+) EGFR(2+).
- Figures:
  1. colon & rectum

手術日期及方法

- 手術日期及方法20210317 Robot Assisted Colectomy(L)

住院治療經過

- After admission, no contraindication was noted for robotic AR. The operation was then performed smoothly on 03/17. However, fever up to 39'C wihout chillness was noted. Tazocin was administered. He tried water on 03/20 and fever was noted again. There was no abdominal pain so abx with NPO was applied. Since no fever was noted , he started tried BLAND II on 03/21. He tolerated well and flatus and stool passage were both noted. Surgical wound was clean and no discharge. Since he was under a relatively stable condition, he was then discharged on 03/23 with oral Curam. OPD f/u was also arranged.

合併症

一般檢查

- 累積報告 :

DATE	TIME	ALB	TP	BIL.T	BIL.D	ALKP	AST	ALT	LDH	CHOL	TG	HDL-C	LDL	UA	NH3	AMY
1100317	1952	3.9		0.4	0.1	67	12	9								
1100319	1045	3.2	5.1	0.53	0.22	47	15	6	176							
1100320	0713			0.7	0.2	50	14	8								
1100322	0933	3.0	5.3	0.53	0.19	81	19	12	179							

DATE	TIME	WBC	RBC	HGB	HCT	MCV	PLT	BLST	NEUT	BAND	SEG	LYM	MON	EOS	BAS
1100317	1951	21790	4.99	14.6	45.5	91.2	258			4.0	90.0	4.0	2.0	0.0	0.0
1100319	1046	15630	4.07	12.3	36.6	89.9	194			7.0	75.0	7.0	9.0	0.0	0.0
1100320	0707	13710	3.88	11.3	35.8	92.3	180		82.2			11.7	5.5	0.5	0.1
1100322	0921	11340	3.90	11.8	34.6	88.7	258		78.7			11.8	7.6	1.7	0.2
1100322	0921								78.7			11.8	7.6	1.7	0.2

DATE	TIME	NA	K	CL	CA	BUN	CREAT	Mg	P	CRP	ESR	PCT	LIPASE	LACTATE
1100317	1952	141	4.0	104	8.7	9	0.83							
1100319	1045	138	3.6	101	7.7	9	0.95							
1100319	1054													8.5
1100320	0706													9.1
1100320	0713	137	3.6		7.7	10	1.02							
1100322	0933	138	3.1	98	7.6	7	0.73							

DATE	TIME	GLUCOSE	PAB	TRF	ZN	CU	KETONE	CK	CKMB	PT-P	PT-C	APTT-P	APTT-C	TRO-T
1100317	1951	163												

DATE	TIME	PH	BEB	SCVO2	PO2	HCO3	HGB	PCO2	TCO2
1100320	0659	7.316	0.6	33.7	22.9	28.0		56.1	29.7

特殊檢查

病理報告

- pending on surgical pathology

其它

出院時情況

- 3.改門診治療

出院指示

藥名	劑量	劑量單位	用法	頻次	數量	數量單位	現況	天數	單位
Curam Film-coated tab 1000mg	1	TAB	PO	Q12H	14	TAB	TH	7	天
K-Glu oral solution 15mL	15	ML	PO	TIDPC	15	ML	TH	5	天
Duo Jardiance tab 12.5/850mg	1	TAB	PO	BID	16	TAB	TH	8	天
Amaryl tab 2mg	1	TAB	PO	BIDAC	16	TAB	TH	8	天
500mg Glucophage	1	TAB	PO	BID	16	TAB	TH	8	天

出院計劃

主治醫師 2062F 蔣鋒帆 / 住院醫師 9757K 翁明謙

正式摘要(電子簽章：**000000212852589)**

W52 / W52

doctorRank:B
